# Effectiveness of Opioid Switching in Advanced Cancer Pain: A Prospective Observational Cohort Study

**DOI:** 10.3390/cancers15143676

**Published:** 2023-07-19

**Authors:** Aaron K. Wong, Andrew A. Somogyi, Justin Rubio, Tien Dung Pham, Brian Le, Pal Klepstad, Jennifer Philip

**Affiliations:** 1 Department of Palliative Care, Peter MacCallum Cancer Centre, Melbourne 3052, Australia; 2 Department of Palliative Care, The Royal Melbourne Hospital, Melbourne 3050, Australia; 3 Department of Medicine, Faculty of Medicine, Dentistry and Health Sciences, The University of Melbourne, Parkville 3052, Australia; 4 Discipline of Pharmacology, Faculty of Health and Medical Sciences, University of Adelaide, Adelaide 5005, Australia; 5 Florey Institute of Neuroscience & Mental Health, Parkville 3050, Australia; 6 Department of Circulation and Medical Imaging, Norwegian University of Science and Technology, NO-7491 Trondheim, Norway; pal.klepstad@ntnu.no

**Keywords:** opioid, analgesics, advanced cancer, palliative care

## Abstract

**Simple Summary:**

Opioid switching is the practice of substituting one opioid for another to improve pain relief or reduce adverse effects. This study aimed to examine pain and adverse event outcomes in people with advanced cancer pain, comparing those who undergo opioid switching with a control group, using multidimensional pain and standardized adverse event recording to add toward the limited data on this common practice. We found that compared to the control group, opioid switching reduced pain (worst, average, now) (*p* < 0.05), uncontrolled breakthrough pain (a 3-fold reduction, *p* = 0.008), and psychological distress (48% to 16%, *p* < 0.005). This study demonstrates that opioid switching is effective at reducing pain, adverse effects, and psychological distress to satisfactory levels of symptom control within 1 week in a population with advanced cancer pain. The use of multidimensional tools and standardized reporting further adds to the quality of evidence supporting opioid switching practice.

**Abstract:**

Opioid switching is a common practice of substituting one opioid for another to improve analgesia or adverse effects; however, it has limited evidence. This study aimed to examine the effectiveness of opioid switching in advanced cancer. This multi-center prospective cohort study recruited patients assessed to switch opioids (opioid switch group) or to continue ongoing opioid treatment (control group). Clinical data (demographics, opioids) and validated instruments (pain and adverse effects) were collected over two timepoints seven days apart. Descriptive analyses were utilized. Non-parametric tests were used to determine differences. Fifty-four participants were recruited (23 control group, 31 switch group). At the follow-up, opioid switching reduced pain (worst, average, and now) (*p* < 0.05), uncontrolled breakthrough pain (3-fold reduction, *p* = 0.008), and psychological distress (48% to 16%, *p* < 0.005). The switch group had a ≥25% reduction in the reported frequency of seven moderate-to-severe adverse effects (score ≥ 4), compared to a reduction in only one adverse effect in the control group. The control group experienced no significant pain differences at the follow-up. Opioid switching is effective at reducing pain, adverse effects, and psychological distress in a population with advanced cancer pain, to levels of satisfactory symptom control in most patients within 1 week.

## 1. Introduction

Pain affects two-thirds of people with advanced cancer [1] and is one of the commonest and most feared cancer symptoms [2,3]. Opioids form the cornerstone of cancer pain management and are generally effective; however, a third of patients with cancer have undertreated pain [4]. Opioid switching, or opioid rotation, occurs where one opioid is substituted with another opioid to either reduce pain, to mitigate intolerable adverse effects (AEs), or for practical reasons (e.g., medication adherence, convenience, or a different preferred route) [5]. Key guidelines on managing cancer pain support this practice [6,7,8], which is now a common and accepted approach to cancer pain management [3].

Observational data suggest opioid switching to be beneficial [5,6,9,10]; however, the rationale and mechanisms underlying the clinically different effects of various opioids in different people have not been well-studied, limiting support for this practice [3,9,11,12,13,14]. Randomized trials comparing opioids such as morphine, oxycodone, hydromorphone, and methadone show no differences in efficacy or tolerability between these opioids; hence, one opioid should not necessarily be superior as the first-line opioid to another [6,15,16,17]. It has been suggested that the observed analgesic improvement following opioid switching is due solely to opioid dose escalation [13], with some data reporting on patients receiving as little as 30 mg oral morphine equivalent per day prior to switching [12,13]. Conversely, other data suggest improved adverse effects may be due to a reduced opioid equivalent dose post-switch. [18]. Systematic reviews on opioid switching [3,13,18] have shown low case numbers (≤32) in many studies [13]. Other issues include the use of retrospective datasets, uncontrolled prospective data, unclear dose conversion schedules, and variations in the report of AEs. Many studies [12,13,18] use a single question to determine pain outcomes (e.g., a numerical rating scale or visual assessment score), which may be too simplistic for the multidimensional evaluation required in this setting [12]. An overview of Cochrane reviews on opioids in cancer pain concluded that the quality of evidence on opioid switching is “very low” [3]. In its 2019 update on cancer pain management, the World Health Organization (WHO) made no specific recommendations supporting or opposing opioid switching, citing a lack of strong evidence in this area [11]. Nevertheless, opioid switching remains common practice [3,6,7,8].

To address the limitations of previous work, this study aimed to compare a cohort of people with cancer pain who undergo opioid switching, with a comparator control group in a prospective longitudinal study. The characteristics of each group are carefully described, with information recorded for cancer type, pain experienced, opioid administered, psychosocial outcomes, adverse effects (AEs), and hematological and biochemical data, to satisfy a multidimensional assessment of the effects of opioid switching compared to those on controls using standardized and validated tools.

## 2. Materials and Methods

Design and Population: This multi-center prospective open cohort study included adult patients with pain from advanced cancer who were known to palliative care services and had received stable doses of slow release (SR) opioids to manage pain for at least three days before enrolment. Participants were recruited into two groups—(1) the Switch Group, in which patients were deemed to require a switch in SR opioids for any reason at enrolment, and the (2) Control Group, which included patients on a stable SR opioid dosing regimen. For those in the switch group, the requirement for an opioid switch was based on the decision of the attending palliative care physician, with reasons for the switch being documented. Patients were included from five tertiary metropolitan hospitals with cancer and palliative care services in Victoria, Australia, from April 2019 to May 2020. The study was approved by the St Vincent’s Hospital Melbourne Ethics Committee (HREC 252/18). All participants provided written informed consent to participate in the study.

Data Collection: Data were collected at the baseline (T0), and again 7 days later (T1) in order to provide adequate time for observation and to reflect plasma opioid steady-state conditions for the switch group. The control group received the same SR opioid at both timepoints; however, the dose was dependent upon clinical requirement at each timepoint (T0 and T1). The switch group received the final dose of their initial SR opioid at T0, after which their SR opioid was switched, and data were collected 7 days later (T1).

The collected data included demographics, clinical and cancer characteristics, the Charlson comorbidity score [19], opioid characteristics (dose, frequency, and route), and concurrent medications. Blood samples (hematology, renal and liver function, DNA, and RNA) were included as part of a larger study examining the pharmacogenomics and pharmacokinetics of opioids; however, these are not reported in this paper. Opioid dose was standardized to the oral morphine equivalent daily dose (oMEDD) using an opioid dose equivalence calculation table [20].

Pain characteristics were assessed using patient-reported questionnaires (Brief Pain Inventory–Short Form (BPI-SF) [21], self-reported Leeds assessment of neuropathic symptoms and signs (S-LANNS) [22], Edmonton Classification System for Cancer Pain (ECS-CP) [23], and Alberta Breakthrough Pain Assessment Tool [24]). Adverse effects were collected using the Edmonton Symptom Assessment System Revised Scale (ESAS-R) [25]. Clinicians assessed cognitive adverse effects using the Nursing Delirium Screening Scale (NUDESC) [26] and Memorial Delirium Assessment Scale (MDAS) [27], and assessed the efficacy of pain treatment using the Clinical Global Impression of Change (CGI-C) [28] instrument. All instruments used were validated. A score of ≥4 on the Edmonton Symptom Assessment Scale was used to define uncontrolled symptoms [25].

Analyses: Descriptive statistics were used to report all measures. Continuous variables were expressed as medians with interquartile ranges (IQR) and categorical variables as counts (N) and percentages. Wilcoxon’s signed-rank test and McNemar’s test were used to determine differences in scores for pain, adverse effects, and associated symptoms between T0 and T1 within both groups. Conditions in which the sample size was fewer than ten discordant pairs were excluded from hypothesis testing as this did not meet the minimum sample requirement for the McNemar test [29]. The mid-p exact test was used to determine differences in the number of patients who experienced moderate–severe adverse effects (≥4 score) for each group between T0 and T1, and between groups at T0, as well as for a comparison of opioid dose increase between T0 and T1. When statistically significant (*p* < 0.05) differences were found, the 95% CI of differences was calculated. An analysis plan was generated a priori to enable the standardized coding of descriptive data into relevant categorical and/or numerical variables for inclusion in the result tables. Median values or scores were used to reduce the effect of outliers. Analysis was undertaken using R Statistical Package (version 4.0) [30].

## 3. Results

The study comprised 54 patients who had cancer-related pain, with 23 control group and 31 switch group participants (Table 1). The median age of the total group was 63.7 years (IQR 55.4–72.9). The commonest primary cancer type was gastrointestinal (25% control; 36% switch), followed by lung (25% control; 32% switch). Participants had an average of two metastatic sites, most commonly bone. Renal and liver function at the baseline were similar across the two groups.

### 3.1. Opioid Data and Switching

At T1, the median opioid dose was higher in the switch group compared to that in the control group. The switch group had an increase in the median dose from T0 to T1 of 72% compared with that of 33% for non-switchers (*p* = 0.41) (Table 1). The most frequently used SR opioid at T0 was oxycodone (n = 28 (52%)) (Table 2). The most prevalent opioids to switch from were oxycodone (n = 15; 48%) and morphine (n = 6; 19%), and those to switch to were hydromorphone (n = 10; 32%) or morphine (n = 10; 32%).

Based on clinician assessment, all patients in the switch cohort underwent opioid switching due to uncontrolled pain, with seven (21%) also having an opioid-related adverse effect, most commonly constipation, followed by nausea with fewer patients reporting itch, hallucinations, sedation and myoclonus.

### 3.2. Pain Assessments and Psychological Distress

Patients who were assessed to be in need of an opioid switch had higher scores for worst pain (8 vs. 6, *p* = 0.008) and more frequent breakthrough pain (three vs. two episodes per day, *p* = 0.005) than patients kept on stable opioid therapy. Post-switch, a statistically significant one-point improvement was observed in almost all of the pain questions (worse, average, and pain now), and also in several domains for the influence of their pain on function (general activity, mood, insomnia, and tiredness). There were also statistically significant reductions in daily breakthrough pain frequency (three vs. two per day, *p* = 0.001) and those experiencing ≥4 breakthroughs per day (45% vs. 14%, *p* = 0.008) (Table 3). Clinician-rated participant psychological distress also reduced in the switch group (48 vs. 16%, *p* < 0.005).

There were no statistically significant (*p* > 0.05) improvements in pain relief percentages and in the non-pain domains. The control group had no statistically significant differences in pain scores between T0 and T1 (Table 3).

### 3.3. Adverse Effects

The frequency of patients with AEs of moderate to severe intensity (score ≥ 4/10) is reported in Table 4.

The commonest uncontrolled patient-reported symptom at the baseline was tiredness (94% switch; 59% control). For each symptom, the switch group had at T0 more participants with uncontrolled symptoms, and these were statistically significant for nausea, tiredness, drowsiness, depression, and itch (*p* < 0.05). The absolute scores of AEs are given in Supplementary Table S1.

In the switch group, participants reported a ≥25% reduction in the frequency of 7 of the reported moderate–severe symptoms at T1 (nausea, tiredness, drowsiness, breathlessness, depression, wellbeing, and constipation), whereas in the control group, only one symptom (anxiety) was reduced by a similar factor. Statistically significant reductions were seen in tiredness in the switch group (32.3% reduction; *p* = 0.003), and anxiety in the control group (50% reduction; *p* = 0.045). The NUDESC and MDAS scales showed that all patients did not have delirium at either timepoint. The Clinical Global Impression of Change is not reported due to missing data.

## 4. Discussion

We observed, as have others [9,10,12,13,18], that the opioid switch group represented a population of patients with difficult pain control. At the baseline, patients in the switch group experienced worse pain (background, breakthrough, and incident pain), more neuropathic pain (a higher S-LANNS score), a greater influence of pain on function (functional items from the BPI), adverse effects (items from ESAS), and psychological distress (one item from ECS-CP). Both groups had similar distributions across cancer type, metastasis site, opioid type at baseline (54% on oxycodone) and similar renal and liver biochemistry. We found that the switch group had significant improvements within 1 week following opioid switching based on a series of measures involving pain scores, breakthrough pain, pain control goals, and adverse effects.

Studies have reported improvements in similar pain score outcomes post-switch. For example, recently, Mercadante et al. [10] found a similar post-switch ESAS pain score reduction to 3.48 at a median of 6 days. Other studies using other scoring systems on a 0–10 scale (e.g., visual assessment scale, independent numerical rating scale) have also found pain outcomes of 3 or less at 3–14 days post-switch [3,13]. The frequency of breakthrough pain has also been used as a surrogate marker of analgesic efficacy, where three to four episodes/day is viewed as acceptable [31,32,33], with a median frequency of three episodes/day [34]. After opioid switching, a 3-fold reduction was seen in uncontrolled breakthrough pain frequency, being reduced from 45% to 14% patients experiencing ≥4× episodes per day, bringing the switch group’s breakthrough pain pattern to the level of that of the control group.

This study defined a pain score of ≤3/10 as controlled pain. This agrees with Hui et al. who in a similar palliative care setting [35] compared ESAS scores and personalized symptom goals (PSGs) where a median ESAS pain rating of three or less was defined as satisfactory [25,35,36]. In our study, the switch group had reductions in ESAS pain scores to three, indicating that their pain was controlled post-switching. This was equal to the control group who were initially identified as having controlled pain, and whose pain remained at three throughout the study [25,37]. Our data thus show statistically significant and clinically meaningful reductions in pain scores across two validated instruments (BPI-SF and ESAS-R), with the reduction in pain being equal to the minimum clinically important difference on the ESAS, and to the level of the PSG for satisfactory pain control. In addition, breakthrough pain outcomes were reduced to acceptable levels under the suggested guideline [9,31,32,34].

In our study, clinicians reported that only 7 (21%) patients were reported to undergo opioid switching due to intolerable AEs, with minimal differences seen post-switch. Similarly, a systematic review found that AE frequency only rarely lessened post-switch, but also pointed out that AEs were inconsistently measured in opioid switching studies. Various studies have reported either a small (25–34%) [38,39,40] or large (>90%) [41,42,43] prevalence of AEs, and this may relate to how AEs were collected and attributed within each study. A lack of opioid pain control without dose-limiting AEs may indicate that the opioid dose is not adequately titrated. Still, experienced palliative care physicians prefer to switch opioids than to increase the dose of the current opioid. This may indicate a reluctance to give high-dose opioids or that the measures of AEs under the NRS in an ESAS questionnaire do not reflect all impressions observed in a physician–patient encounter.

The degree of association between each adverse effect and opioids can be difficult to measure, as patients with advanced cancer can have multiple, inter-related and multifactorial symptoms. Symptoms may be caused by the cancer itself, anti-cancer treatments, other chronic non-cancer conditions and opioids. Some studies have included any patient-reported symptom that could possibly be an opioid AE (e.g., nausea, drowsiness, constipation, and fatigue) as opioid-related [10,41,43]. Almost every patient in the control group and every patient in the switch group had at least one uncontrolled symptom on the ESAS, (score ≥ 4) that could be attributable to opioids. At the follow-up timepoint, the switch group had a ≥25% reduction in seven uncontrolled symptoms post-switch (nausea, tiredness, drowsiness, breathlessness, depression, wellbeing, and constipation), as opposed to only one symptom in the control group (anxiety). Similarly, the switch group started with eight of the nine reported ESAS symptoms being above the PSG [35], which post-switch halved to only four symptoms at this severity level, which is the same as that of the control group at the follow-up. This supports the notion that opioids have a major role in impacting various non-pain symptoms. The attribution of AEs requires careful consideration of both clinician and patient opinions.

We recognize several limitations to this study. First, it was unblinded and the patients were not randomized. Still, the observations reflect daily clinical practice in the selection of patients for an opioid switch and the results from the switch. Second, a larger sample size would have afforded more statistical power to determine definitive associations and conduct subgroup analyses for various opioid switch pairs on the degree of pain type (e.g., nociceptive vs. neuropathic pain), oMEDD, and AEs. Nevertheless, our sample of 58 participants in total is larger than that of most studies on opioid switching and has demonstrated statistically significant differences across various clinically important pain and non-pain outcomes. Finally, other factors than the opioid switch may have influenced changes in the seven-day follow-up period. However, since the time interval between timepoints was only 7 days, it is likely that these symptoms improved as a result of opioid switching. This is supported by the presence of stable symptoms in the control group.

## 5. Conclusions

This study shows that opioid switching is effective for a population of cancer patients undergoing palliative care who have severe pain that is refractory to their current opioid use. Opioid switching improved background and breakthrough pain to satisfactory control levels within 1 week. This study also assessed and found improvements in multiple dimensions related to pain, namely psychological distress, AEs, and functional ability scores which improved to levels similar to those of patients who were comfortable on their baseline dose of an opioid (the control group).

Future directions should include the ascertainment of larger patient cohorts and a focus on more homogeneous patient groups in a smaller number of opioid types. Sub-group analyses would then be powered to investigate different opioid switch pairs to investigate reasons for certain opioids being preferred for certain groups of people. A longer follow-up period could determine the degree of long-term success of opioid switching, and whether or not the strength of its effectiveness remains over subsequent switches, which could allow for the pre-emptive “prognostication” of the likely requirement for opioid switching, which may alter how closely these patients are clinically monitored. The addition of pharmacokinetic and genomic testing will further strengthen the ability to carry this out. A standardized assessment of AEs that combines both clinician and patient-reported outcomes may reduce the large variability in AE reporting.

## Figures and Tables

**Table 1:** Participant demographics, opioid dose, biochemistry, and hematology results for opioid switch and control groups.

Characteristic	Control Group (n = 23)	Switch Group (n = 31)
Male Gender, n (%)	12 (50)	20 (59)
Age (years), median (IQR)	69 (60.5–75.5)	61 (49–70)
Australian Karnofsky Performance Score (AKPS), median (IQR)	50 (40–60)	60 (50–70)
Charlson Comorbidity Index Score, median (IQR)	9 (8–9.5)	8 (6–9)
Average metastasis sites per patient	2	2
**Cancer Type**	**n (%)**	**n (%)**
Lung	6 (25)	10 (32)
Breast	5 (21)	2 (6)
Upper GIT	5 (21)	4 (13)
Lower GIT	1 (4)	7 (23)
Prostate	3 (13)	2 (6)
Head and Neck	0 (0)	3 (10)
Other (primary CNS, hematological, bone/soft tissue, urological, and adenocarcinoma of unknown primary)	4 (17)	3 (10)
Metastases Sites *	**n (%)**	**n (%)**
Bone	13 (54)	19 (56)
Lymph notes	7 (29)	13 (38)
Liver	8 (33)	9 (26)
Lung	5 (21)	5 (15)
Central nervous system	5 (21)	3 (9)
Peritoneal	1 (4)	6 (18)
Soft tissue	1 (4)	2 (6)
Other/unknown	3 (13)	4 (12)
Total metastasis sites	43	61
**Previous Cancer Treatments**	**n (%)**	**n (%)**
Surgery	8 (33)	15 (44)
Radiotherapy	18 (75)	24 (71)
Systemic therapy (e.g., chemotherapy, immunotherapy, targeted therapy, and hormonal therapy)	18 (75)	25 (74)
None	2 (8)	2 (6)
**Blood Results**	**Median (IQR)**	**Median (IQR)**
eGFR (mL/min/1.73 m^2^)	90 (84.5–90)	90 (87–90)
Albumin (g/L)	28 (23.5–33)	30 (23–34)
GGT (IU/L)	105 (32–285)	64 (28–119)
AST (IU/L)	41.5 (28–53)	21 (18–31)
ALT (IU/L)	28 (19–48)	17 (11–32)
CRP (mg/L)	55 (19.1–126.5)	72.7 (30.8–108)
Hb (g/L)	105 (92–112)	105.5 (94–117)
WCC (x109/L)	8.4 (5.7–10.7)	8.9 (6.3–11.2)
	**T0 T1**	**T0 T1**
**Total opioid dose last 24 h (mg), median (IQR)**	135 180 (60–230) (67.5–300)	120 207.5 (65–240) (80–348.8)

* Each participant can have >1 metastasis sites.

**Table 2:** Slow-release opioid for control group and switch group at T0 and T1.

	T0, Control Group (n, %)	T0, Switch Group (n, %)	T1, Switch Group (n)				
			Hydromorphone	Morphine	Oxycodone	Fentanyl	Buprenorphine
Oxycodone	13 (56)	15 (48)	4	8	-	3	-
Morphine	4 (17)	6 (19)	3	-	2	1	-
Buprenorphine	1 (4)	4 (13)	2	1	-	1	-
Fentanyl	1 (4)	4 (13)	1	1	1	-	1
Hydromorphone	4 (17)	1 (3)	-	-	1	-	-
Tramadol	-	1 (3)	-	-	1	-	-
Total	23	31	10	10	5	5	1

**Table 3:** Comparison of questionnaire responses to pain and other symptoms across opioid switch and control groups at T0 and T1.

ECS-CP *		**Control**			**Switch**		
	**Item**	**T0—n (%)**	**T1—n (%)**	***p* Value**	**T0—n (%)**	**T1—n (%)**	***p* Value**
	Q1 Neuropathic pain	4 (19)	4 (19)	***	16 (48)	14 (45)	0.66
	Nociceptive pain	17 (81)	14 (78)	0.34	17 (52)	17 (55)	0.66
	Q2 Incident pain present	11 (52)	7 (39)	0.16	21 (64)	20 (65)	0.71
	Q3 Psychological distress	7 (33)	6 (33)	***	16 (48)	5 (16)	<0.005
	Q4 No cognitive impairment	19 (90)	18 (100)	***	31 (94)	30 (97)	0.56
	Q5 No addictive behavior	18 (86)	15 (83)	0.18	33 (100)	30 (97)	***
	Total, n	21	18	-	33	31	-
S-LANNS Score > 12		4/21 (19%)		-	13/31 (42%)		-
**Brief Pain Inventory—Short Form (BPI-SF)**		**Control**			**Switch**		
	**Symptom**	**T0 Median (IQR)**	**T1 Median (IQR)**	***p* Value**	**T0 Median (IQR)**	**T1 Median (IQR)**	***p* Value**
	Worst	6 (4, 8)	6 (5, 7)	0.55	8 (7, 9)	7 (3.3, 8)	<0.005
	Least	1 (1, 3)	2 (0.3, 2.8)	0.96	2 (1, 4)	2 (0.3, 3)	<0.01
	Average	4 (3, 5)	4 (2.3, 5)	0.63	5 (4, 6)	4 (2.3, 5)	<0.005
	Now	2 (0, 4)	3 (1.3, 4)	0.40	4 (3, 6)	3 (1,4. 75)	<0.005
	Pain Relief percentage	80 (50, 100)	70 (50, 100)	0.96	60 (25, 80)	80 (50, 90)	0.07
	General activity	4 (2, 7)	6 (3, 9)	0.35	7 (4.5, 9)	4.5 (1.3, 7.8)	<0.05
	Mood	2 (0, 5)	2 (0, 5)	0.62	7 (4.5, 8)	5 (0.3, 7)	<0.05
	Insomnia	3 (0, 6)	2 (0, 5)	0.75	7 (3.5, 8.5)	4 (1, 8)	<0.05
	Walking ability	6 (2, 8)	6 (0, 10)	0.88	6 (2, 8)	5 (0.3, 8)	0.51
	Normal work	5.5 (1.5, 9)	8 (0, 9)	0.89	8 (3.3, 9)	5.5 (1.8, 8)	0.33
	Relations with others	0 (0, 4)	0 (0, 5)	0.34	7 (3, 8)	5 (0.3, 7)	0.17
	Enjoyment of life	6 (2, 8)	8 (3, 9)	0.50	8 (6, 9)	5 (2, 9)	0.073
ABPAT **		**Control**			**Switch**		
		**T0**	**T1**	***p* value**	**T0**	**T1**	***p* value**
	Breakthrough pain frequency, median (IQR)	2 (1, 3) (n = 17)	2 (1, 2) (n = 17)	0.275	3 (3, 6) (n = 22)	2 (0, 3) (n = 22)	0.001
	Breakthrough episodes ≥4/day, n (%)	2 (12%)	2 (12%)	>0.99	10 (45%)	3 (14%)	0.008

* Edmonton Classification System for Cancer Pain Scale (items with 2 or less responses are excluded); ** ABPAT (Alberta Breakthrough Pain Assessment Tool); *** Conditions in which the sample size was fewer than ten discordant pairs were excluded from hypothesis testing as this did not meet the minimum sample requirement for the McNemar test.

**Table 4:** Comparison of frequency of participants in the opioid switch and control groups with moderate–severe symptom scores (≥4 score) for each symptom.

	Control		Difference	*p* Value	Switch		Difference	*p* Value	Control T0 vs. Switch T0 *p* Value
	T0 (n = 22)	T1 (n = 23)	T1-T0 (n, %)		T0 (n = 33)	T1 (n = 31)	T1-T0 (n, %)		
Nausea	3	2	−1	0.64	13	7	−6 (−46.2)	0.08	<0.05
Constipation	7	5	−2	0.24	13	8	−5 (−38.5)	0.13	0.29
Tiredness	13	13	0	0.50	31	21	−10 (−32.3)	0.004	0.001
Drowsiness	11	10	−1	0.34	24	18	−6 (−25.0)	0.12	0.05
Depression	6	6	0	0.47	18	12	−6 (−33.4)	0.11	<0.05
Anxiety	10	5	−5 (−50.0)	0.045	13	10	−3 (−23.1)	0.28	0.33
Wellbeing	14	13	−1	0.32	25	18	−7 (−28.0)	0.07	0.18
Itch	1	2	1	0.32	7	9	2	0.05	0.05
Hallucinations	1	0	−1	0.24	3	5	2	0.21	0.30
Hiccups	1	1	0	0.49	5	5	0	0.46	0.13
Total Reported	67	57	−10	0.08	152	113	39	<0.005	<0.001

% only available for difference in >2 participants.

## Data Availability

The data presented in this study are available on request from the corresponding author. The data are not publicly available due to privacy reasons.

## Supplementary Materials

The following supporting information can be downloaded at https://www.mdpi.com/article/10.3390/cancers15143676/s1. Table S1. Comparison of adverse event scores across opioid switch and control groups at T0 and T1.
