# Supplementary material for: Effectiveness of Opioid Switching in Advanced Cancer Pain: A Prospective Observational Cohort Study
Source: Cancers (Basel). 2023 Jul 19;15(14):3676. doi: 10.3390/cancers15143676 (PMC10378198; doi:10.3390/cancers15143676)
Supplement: Supplementary file 1 [file cancers-15-03676-s001.zip › cancers-2497470-supplementary.pdf]

Edmonton Symptom Assessment Scale (ESAS)			Control			Switch		
	**Items**	**PSG ***	**T0 Median (IQR)**	**T1 Median (IQR)**	**P value**	**T0 Median (IQR)**	**T1 Median (IQR)**	**P value**
	**Pain**	**3**	3 (1,5)	3 (2,6)	1	4 (2.3,6.8)	3 (2,5)	<0.05
	**Tiredness**	**3**	6 (3,8)	4 (3.3,8)	0.70	7 (5.5,8)	6 (3,8)	<0.05
	**Drowsiness**	**2**	4 (2,7)	4 (2,7)	0.55	6 (4,8)	4 (2,7)	0.18
	**Nausea**	**1**	1 (1,1)	1 (1,1)	0.21	2 (1,4)	1 (1,3)	0.28
	**Appetite**	**3**	1 (1,5)	1.5 (1,4.8)	0.15	4 (2.3,8)	5 (1,7)	0.68
	**Shortness of Breath**	**2**	1 (1,6.3)	1 (1,4.75)	0.34	4 (1,5)	2 (1,7)	0.97
	**Depression**	**2**	1 (1,5)	1.5 (1,5)	0.55	4 (2,6.8)	2 (1,5)	0.23
	**Anxiety**	**2**	3 (1,5)	1.5 (1,4.5)	0.12	2 (1,5.8)	2 (1,5)	0.78
	**Wellbeing**	**2**	5 (3,7)	5 (2.8,7)	0.76	5 (4,5.5)	5 (3,6)	0.70

**PSG = Personalised Symptom Goal*

Supplementary Table S1: Comparison of Adverse Event scores Across Opioid Switch and Control Groups at T0 and T1
